# A large-scale pedigree resource of wheat reveals evidence for adaptation and selection by breeders

**DOI:** 10.1371/journal.pbio.3000071

**Published:** 2019-02-28

**Authors:** Nick Fradgley, Keith A. Gardner, James Cockram, James Elderfield, John M. Hickey, Phil Howell, Robert Jackson, Ian J. Mackay

**Affiliations:** 1 The John Bingham Laboratory, NIAB, Cambridge, United Kingdom; 2 The Roslin Institute, University of Edinburgh, Easter Bush, Midlothian, United Kingdom; Institute of Science and Technology Austria (IST Austria), AUSTRIA

## Abstract

Information on crop pedigrees can be used to help maximise genetic gain in crop breeding and allow efficient management of genetic resources. We present a pedigree resource of 2,657 wheat (*Triticum aestivum* L.) genotypes originating from 38 countries, representing more than a century of breeding and variety development. Visualisation of the pedigree enables illustration of the key developments in United Kingdom wheat breeding, highlights the wide genetic background of the UK wheat gene pool, and facilitates tracing the origin of beneficial alleles. A relatively high correlation between pedigree- and marker-based kinship coefficients was found, which validated the pedigree and enabled identification of errors in the pedigree or marker data. Using simulations with a combination of pedigree and genotype data, we found evidence for significant effects of selection by breeders. Within crosses, genotypes are often more closely related than expected by simulations to one of the parents, which indicates selection for favourable alleles during the breeding process. Selection across the pedigree was demonstrated on a subset of the pedigree in which 110 genotyped varieties released before the year 2000 were used to simulate the distribution of marker alleles of 45 genotyped varieties released after the year 2000, in the absence of selection. Allelic diversity in the 45 varieties was found to deviate significantly from the simulated distributions at a number of loci, indicating regions under selection over this period. The identification of one of these regions as coinciding with a strong yield component quantitative trait locus (QTL) highlights both the potential of the remaining loci as wheat breeding targets for further investigation, as well as the utility of this pedigree-based methodology to identify important breeding targets in other crops. Further evidence for selection was found as greater linkage disequilibrium (LD) for observed versus simulated genotypes within all chromosomes. This difference was greater at shorter genetic distances, indicating that breeder selections have conserved beneficial linkage blocks. Collectively, this work highlights the benefits of generating detailed pedigree resources for crop species. The wheat pedigree database developed here represents a valuable community resource and will be updated as new varieties are released at https://www.niab.com/pages/id/501/UK_Wheat_varieties_Pedigree.

## Author summary

Breeding activities undertaken in the world’s most important crop species have resulted in large increases in yield potential over the last century. Bread wheat is a key crop for both human and animal nutrition worldwide. To help inform future breeding and research activities, we have developed a pedigree resource of over 2,600 bread wheat accessions, originating from 38 countries and representing more than a century of breeding and variety development. Pedigree-based relationships between lines are largely confirmed by genetic marker data. By combining the genetic and pedigree data sets, we are able to identify genetic signatures of selection across the pedigree, identifying genomic regions selected for via modern breeding activities. The resource developed here will serve as an evolving platform to inform and manage wheat genetic diversity in breeding programmes around the world and highlights the utility of developing and exploiting similar resources in other crop species.

## Introduction

Information of variety pedigree (i.e., ancestry or genealogy) can be used by breeders to prioritise crosses between highly performing parents whilst maintaining genetic diversity in the offspring for selection. However, development of varieties by commercial breeding companies in recent decades may have resulted in knowledge fragmentation and duplication of resources [1]. We propose that an integrated large-scale wheat (*Triticum aestivum* L.) pedigree would be a valuable resource for the wheat research and breeding communities. Its development would allow inheritance and origins of beneficial genes and alleles to be tracked through the pedigree to identify sources of traits and genetic variation for research and efficient exploitation. For example, as new races of pathogens evolve to overcome variety resistances [2], sources of resistance could be quickly identified and integrated into breeding programmes. Where genotype data are available on ancestors in a pedigree, genetic identity or estimated breeding values of ungenotyped descendants could be inferred by pedigree based simulations [3]. Within breeding programmes, selection of breeding material can optimise maintenance of genetic diversity with improvement in breeding value [4]. Crosses between genetically distant parents may present a wider genetic variance available for selection [5] and also result in greater potential for heterosis and higher performance of F_1_ hybrid varieties [6–8]. Information on relatedness among available varieties could also help farmers increase genetic diversity at a farm scale, resulting in resilient systems to deal with climate instability and biotic stresses [9].

Studies of smaller scale pedigrees of crop varieties have often compared calculations of kinship between varieties based on pedigree or genetic marker data. However, these studies commonly used low marker numbers and found low correlations between the two methods for estimating kinship [10–12]. Such studies quickly become outdated: new varieties are released every year, and recent developments in genotyping technologies have meant much higher marker numbers are now available. Other limitations of this comparison include the assumption that a pedigree-based estimation of kinship assumes random inheritance and the absence of selection, which is unlikely to be the case in crop species such as wheat.

Large-scale pedigree databases have been developed as research tools in other crops, including oats [13] and rice [14]. Although the International Maize & Wheat Improvement Center (CIMMYT) hosts a large wheat database, including wheat pedigree data (http://wheatpedigree.net/), this pedigree information is only available on a ‘per accession’ basis. Few other pedigree resources are available in wheat. Here, we present a large-scale pedigree of United Kingdom (UK) wheat varieties and their ancestors, available in a format suitable for visualisation in software such as Helium (The James Hutton Institute, Scotland, UK) [15], Pedigree Viewer (University of New England, Biddeford, ME) [16], and Pedimap (Wageningen University, Wageningen, Netherlands) [17], representing a valuable resource for the wheat-breeding and research community. We validate the pedigree using a subset of 450 genotyped individuals to compare kinship coefficients calculated by markers and pedigree. We demonstrate evidence of breeder selection by comparison of observed genotype distributions with predictions generated via gene dropping simulations under Mendelian sampling of known founder genotypes [18–19]. We show that (i) kinship coefficients calculated from markers or pedigree data show strong positive correlation and that large deviations from this correlation are due to erroneous pedigree or seed source data; (ii) within crosses, selection by breeders favours genetic material from the superior parent to which the selected variety will be disproportionally related; and (iii) higher than expected linkage disequilibrium (LD) in recent varieties and changes in allelic diversity provide evidence of selection by breeders over multiple generations of the pedigree. Details of the lines and genomic regions involved provide insight into selection targets and breeder strategies, and the approaches presented are applicable to many crop species.

## Results

**Fig 1:**
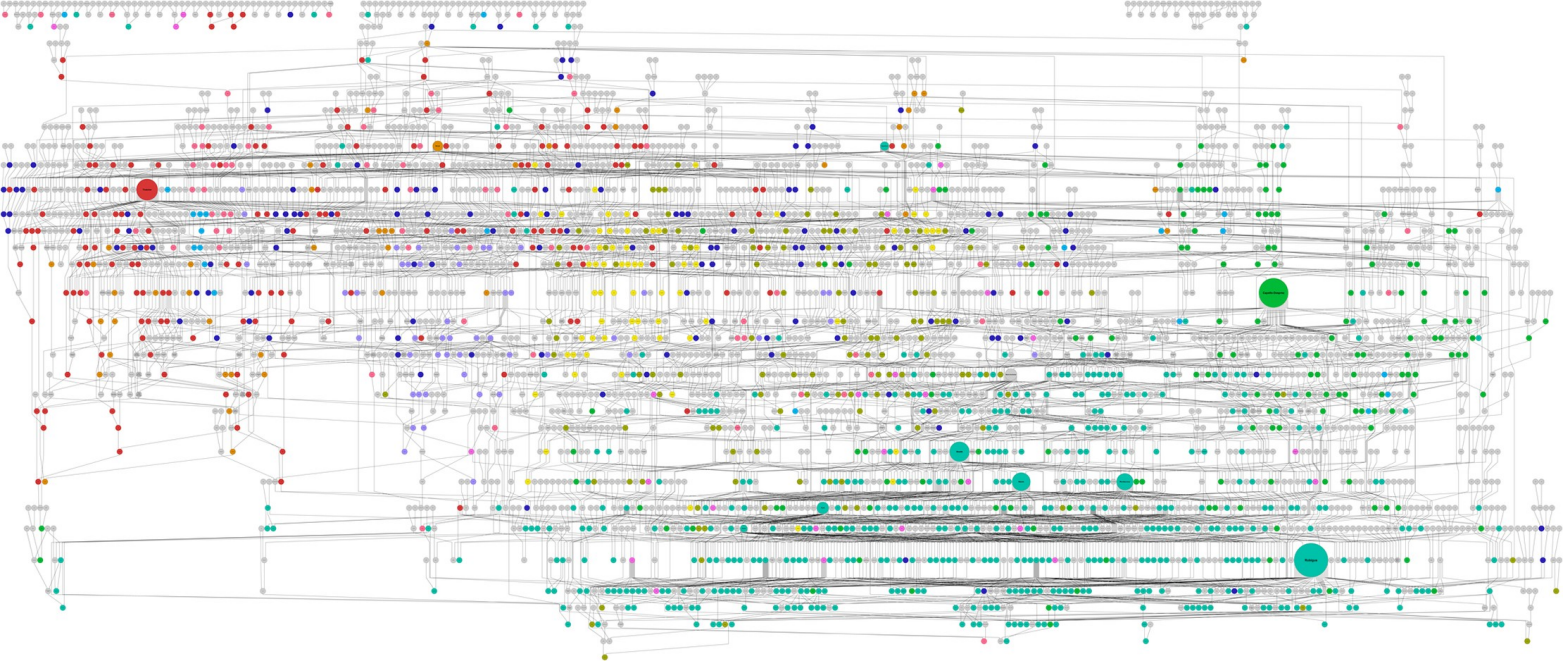
Graphical display of the wheat pedigree, including 2,657 genotypes. Node colour indicates country of origin. Size of each node is proportional to the number of direct offspring. Colour coding: pink (Australia), orange (Canada), light green (Germany), bright green (France), turquoise (UK), light blue (Italy), purple (Mexico), fuschia (the Netherlands), yellow (Sweden), red (US), dark blue (other countries). For a higher resolution image, see S1 Fig.

**Fig 2:**
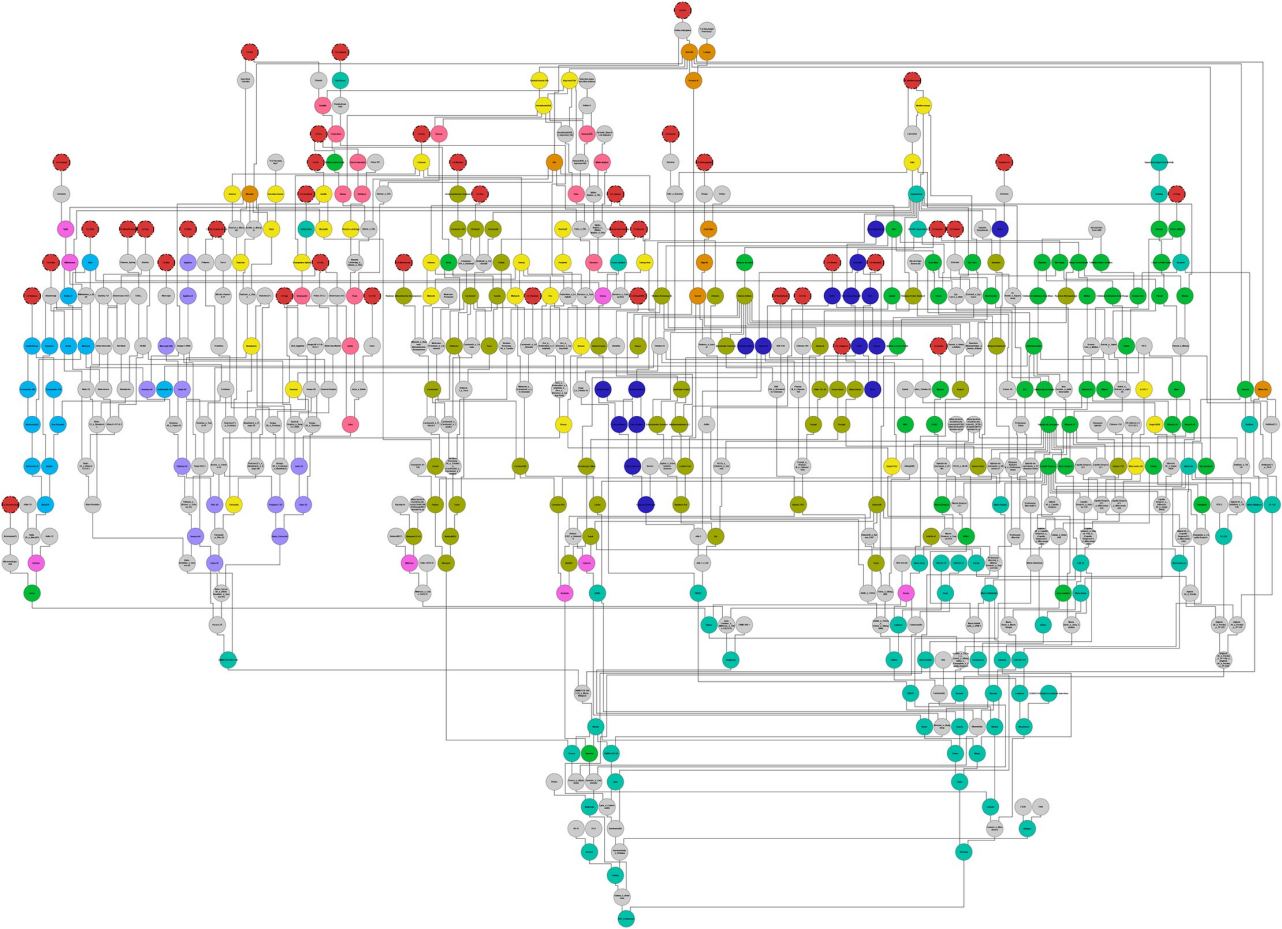
The pedigree of the recent variety ‘RGT Conversion’ (released in 2015, located at the bottom of the image), illustrating the complexity and diversity of the geneology of modern wheat varieties and highlighting the prominence of landraces (Red with dashed outline), as well as the old variety ‘Red Fife’ (a selection from a landrace), at multiple stages within its early pedigree. Colour coding: pink (Australia), orange (Canada), dark green (Germany), bright green (France), turquoise (UK), light blue (Italy), purple (Mexico), fuschia (the Netherlands), dark blue (Sweden), yellow (US), red (landraces). For a higher resolution image, see S3 Fig.

A database of wheat pedigrees was developed that includes a total of 2,657 genotypes originating from 38 countries (S1 Table; also available at https://www.niab.com/pages/id/501/UK_Wheat_varieties_Pedigree), allowing visualisation of the pedigree structure (Fig 1; see also S1 Fig). These include registered crop varieties and intermediate genotypes used in the breeding process, as well as accessions used as early breeding material or selections from heterogeneous landrace material. The extent of the pedigree includes varieties released in the UK up to 2017 and entries as far back as landraces with undetermined origins. Although the majority of varieties were released in the 20th century, 63 varieties were released before 1900. The 1990s represented the decade with the greatest number of varieties released. The varieties most commonly used as parents include ‘Capelle Desprez’ (released in France in 1946 and used 45 times), ‘Thatcher’ (United States 1934, used 41 times), ‘Moulin’ (used 30 times) and ‘Rendezvous’ (used 27 times) (both released in the UK in 1985), ‘Haven’ (UK 1988, used 28 times), and ‘Robigus’ (UK 2000, used 35 times). Although not used directly as a parent currently, a few key historical varieties often feature in the ancestry of more modern varieties. Of the 182 UK varieties release after the year 2000, 89% are known to include both the spring wheat variety ‘Red Fife’ (which originated from a Ukrainian landrace called ‘Ostka-Galicyjska’; See S2 Fig) and ‘Squarehead’ (an early selection from a Mediterranean landrace) in their early ancestries. Additionally, the pedigree allows the complex genealogy of modern varieties to be rapidly analysed. For example, ‘RGT Conversion’ (released in 2015) contains 701 known ancestors, of which 42 are landraces (Fig 2; see also S3 Fig).

### Kinship coefficients

**Fig 3:**
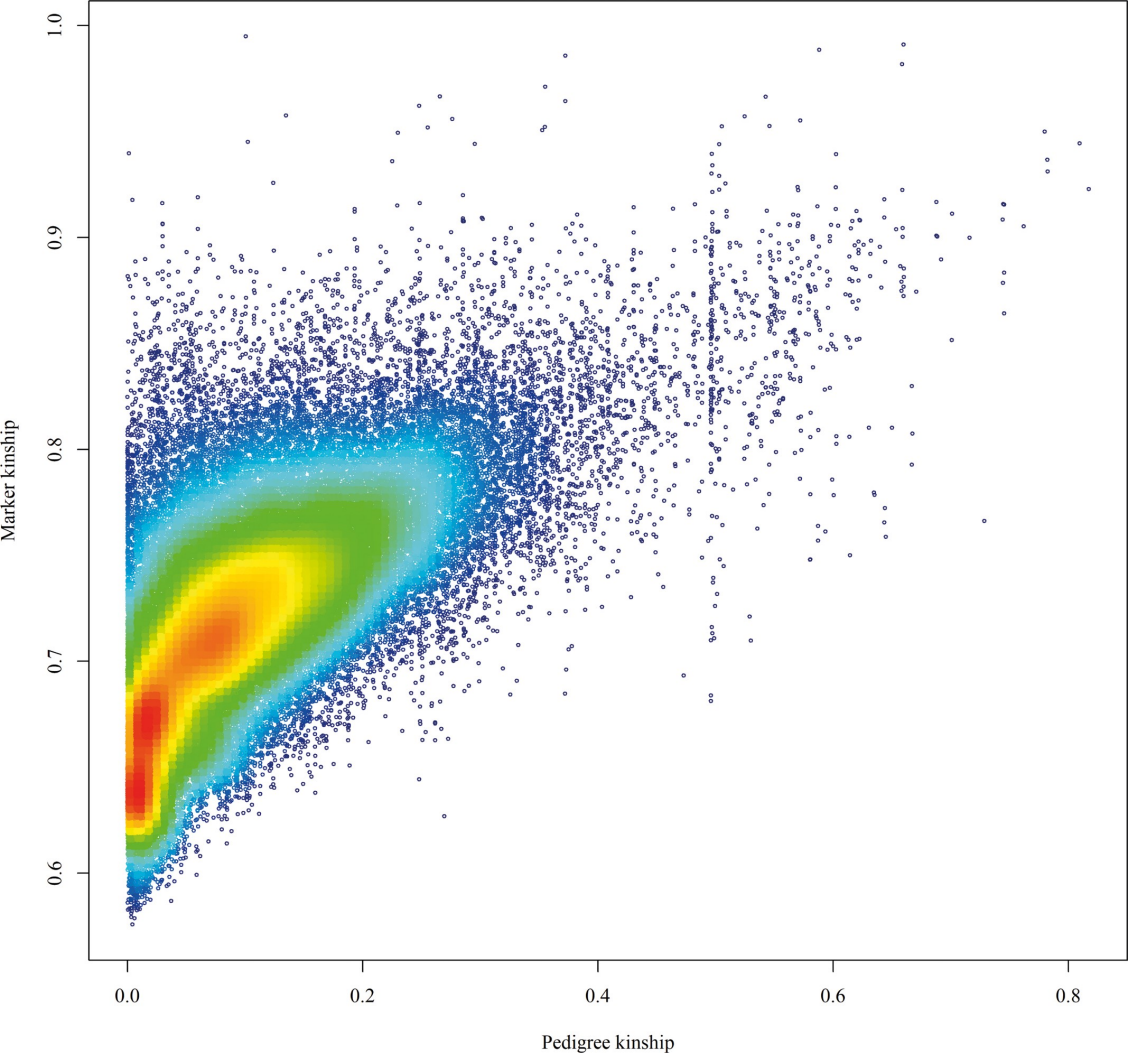
Relationship between genetic and pedigree based kinships coefficients amongst 409 wheat varieties using 4,009 SNP markers. The dark blue to dark red colour coding indicates increasing data point density. SNP, single nucleotide polymorphism.

To account most effectively for multiple generations of inbreeding used in wheat variety development, kinship calculations were made using an augmented pedigree, including seven intermediate generations of selfing from parents to progeny for each accession with parent information. Using the 15,852 entries within the augmented pedigree, 121,391,571 pairwise comparisons of kinship were calculated; 29% of these comparisons were between entries without known common ancestors in the pedigree that gave a kinship of zero and were omitted from further analysis. Pedigree-based kinship coefficients were compared with a subset of 454 varieties for which genotypic data (4,009 single nucleotide polymorphisms [SNPs]) were available. For these, kinship between different varieties based on pedigree varied from 4.7 × 10^−7^ to 0.82 and averaged 0.11, whereas kinships calculated by SNPs varied from 0.58 to 0.99 and averaged 0.72. Although different ranges of kinship values are expected due to differences in the methods used to calculate, a significant correlation (r = 0.63, *P* < 0.001) was found between the two equivalent kinship matrices. Notable, nonrandom deviations from this relationship were found, which facilitated identification of erroneous pedigree information, errors in genotyping, or evidence of strong selection within crosses. Detailed investigation of marker kinships between varieties and their immediate ancestors and descendants revealed clearly erroneous information in either the pedigree or seed source used for genotyping for 40 varieties. When these varieties were removed, an improved correlation of 0.68 was found between marker- and pedigree-based kinships. The removal of five additional closely related varieties with information on only one parent improved this correlation to 0.71. Detection of pedigree and/or seed errors is exemplified in S4 Fig, in which there is a notably disproportionate number of marker kinships of approximately 0.63, which were underestimated based on pedigree information. This anomaly is entirely explained by two lines (‘Cyber’ and ‘Maris Ensign’), which were mislabelled as spring wheat varieties when they were actually more distantly related winter wheat varieties. The relationship between the pedigree and marker kinship estimates for the 409 lines remaining after removal of all identified lines with erroneous information and missing parental data is shown in Fig 3.

### Selection within families

**Fig 4:**
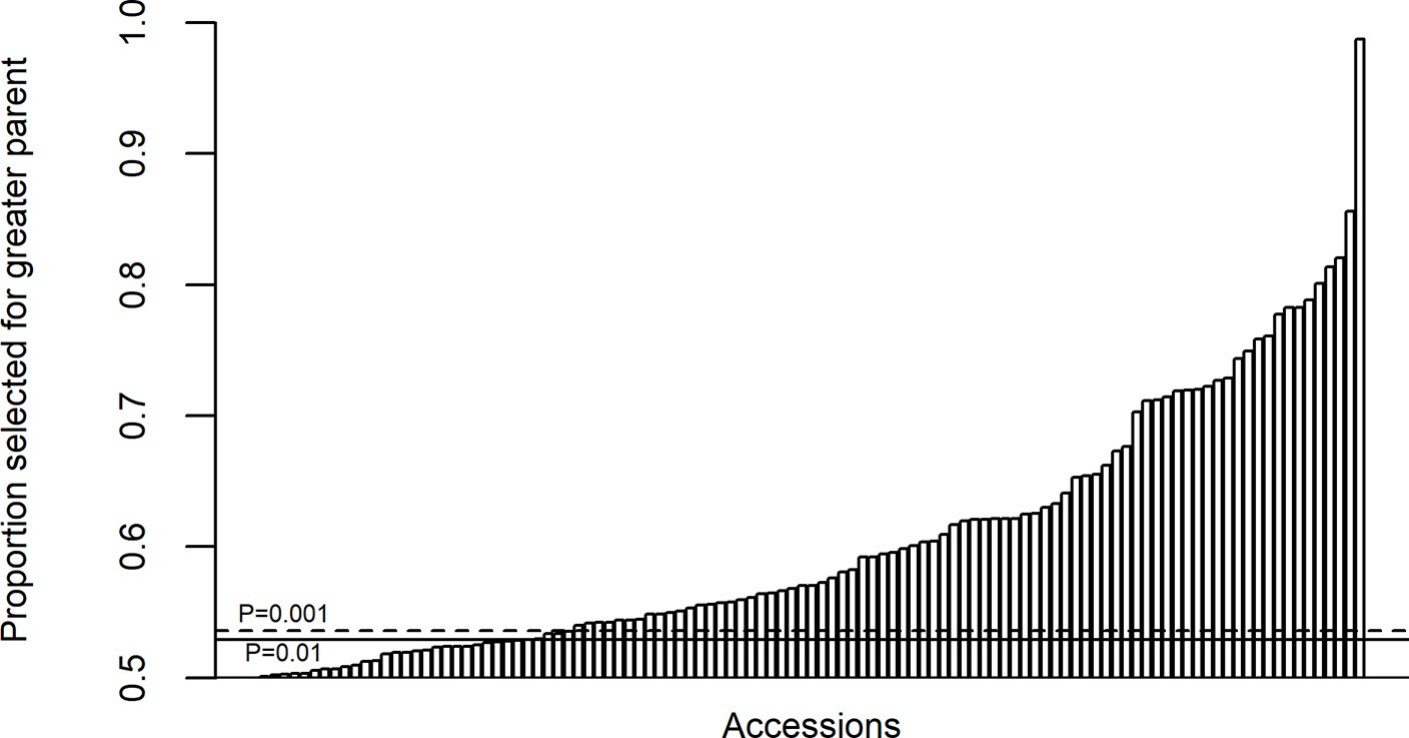
Distribution of allele sharing between progeny line and the maximally related parent for a set of 109 wheat varieties forming simplex families (in which a simplex family represents two parents and their one progeny). Relatedness is based on genotypic data for 4,009 SNPs. Horizontal solid and dashed lines indicate significance thresholds estimated from 1,000 gene dropping simulations. SNP, single nucleotide polymorphism.

Evidence for selection by breeders within family crosses was found using a combination of pedigree and genetic marker data. Out of a set of 109 ‘simplex families’ with SNP data (in which a simplex family represents two parents and their one progeny), the most closely related parent to the progeny within each family shared a median proportion of genome of 0.57, with 77/109 to a greater extent than expected based on 1,000 simulations with gene dropping at the *p* ≤ 0.001 significance threshold of 0.54 (Fig 4). The varieties ‘Robigus’ and ‘Capelle Desprez’ were the most commonly used parents in the pedigree and were always the parent that was more closely related to the progeny, and therefore positively selected, in the five and seven families in which they were included, respectively.

### Selection across the pedigree

**Fig 5:**
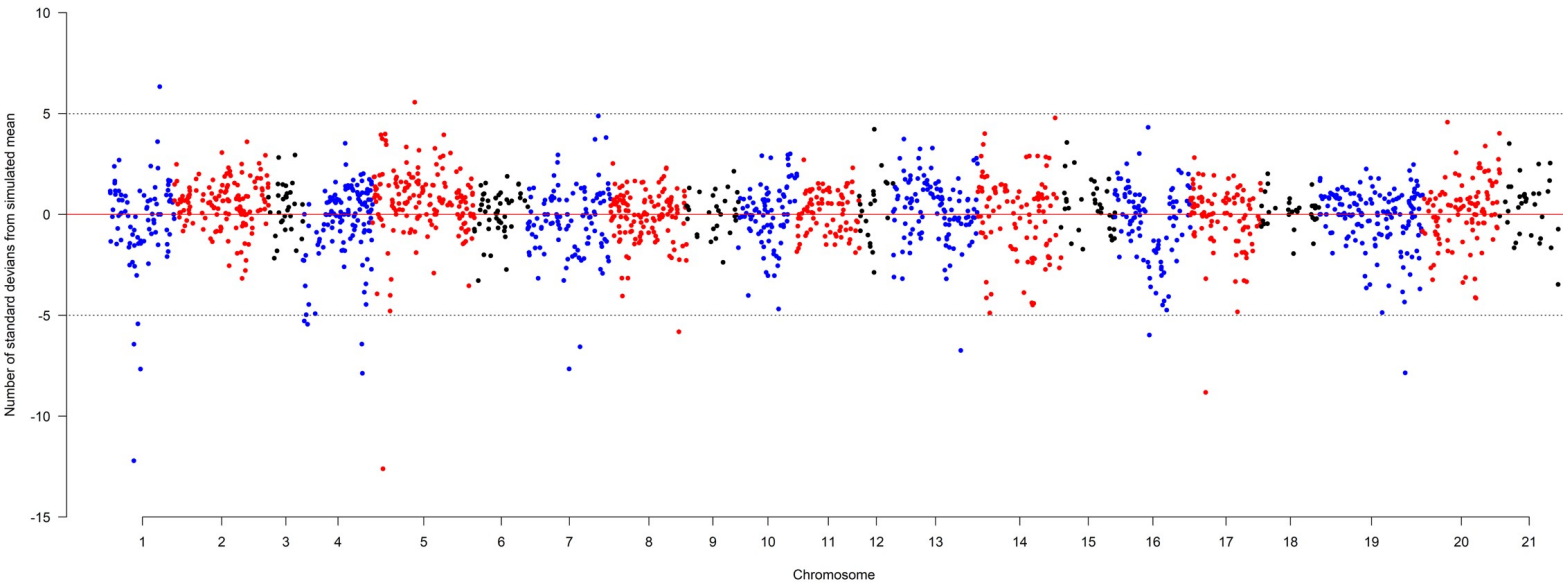
**Difference in SNP allelic diversity across the 21 chromosomes of wheat (from 1A to 7D, with 1 = chromosome 1A, 2 = 1B, 3 = 1D, etc.), indicating magnitude of selection at 1,821 marker positions across the genome.** Simulated data were generated from 100 gene dropping simulations, and observed allelic diversity was compared against simulated distributions for each marker on the y-axis. Horizontal dashed lines indicate five SD thresholds (which approximate to the deviation from the simulated distribution threshold at a Bonferroni corrected *P* = 0.001). SD, standard deviation; SNP, single nucleotide polymorphism.

Additional long-term evidence for breeders’ selection was found by investigating selection effects across the whole pedigree. Using a subset of the pedigree that included just those founders with genotypic information (110), gene dropping simulations compared observed- and simulated-allelic diversity measures for 45 derived varieties released after the year 2000 (S5 Fig). Average values of allelic diversity across all 1,821 SNPs were similar between observed (0.283) and simulated (0.278) genotypes. However, when considering each marker separately, 0.9% of the markers showed a lower diversity than five standard deviations of the simulated distribution (equivalent to a Bonferroni corrected *P* value of 0.001), compared to only 0.1% showing higher diversity than five standard deviations of the simulated distribution found from 100 gene dropping simulations (Fig 5; Table 1).

**Table 1:** Chromosome location and positions of SNPs in linkage with genomic regions under selection, measured by changes in allelic diversity (threshold used: ± five standard deviations).

Chromosome	Position on MAGIC genetic map (cM)^†^	SNP name	Standard deviations from simulated mean
1A	88.3	BS00031066_51	−12.2
1A	88.8	BS00021714_51	−6.4
1A	103.2	IACX742	−5.4
1A	112.8	TA004265_0757	−7.7
1A	184.8	BS00032825_51	6.3
2A	5.6	wsnp_Ku_c33374_42877546	−5.3
2A	18.0	Excalibur_rep_c110303_320	−5.4
2A	219.9	BS00000250_51	−6.4
2A	221.9	BS00023202_51	−7.9
2B	39.4	BS00064706_51	−12.6
2B	157.7	BS00102480_51	5.6
3A	153.9	wsnp_BE494474A_Ta_2_2	−7.7
3A	194.7	BS00038663_51	−6.6
3B	254.8	BS00022715_51	−5.8
5A	252.2	Tdurum_contig13810_485	−6.7
6A	128.4	BS00012297_51	−6.0
6B	57.0	BobWhite_c10239_519	−8.8
7A	321.9	IAAV5268	−7.9

^†^[20]

**Abbreviation:** MAGIC, Multi-parent Advanced Generation Inter-Cross; SNP, single nucleotide polymorphism.

**Fig 6:**
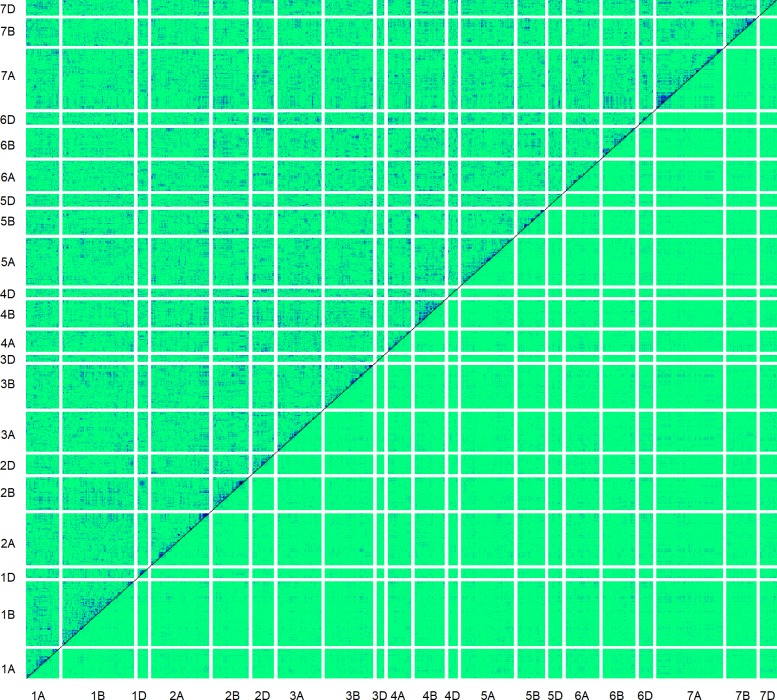
Heat map of LD (R^2^) among 790 SNP markers across the 21 chromosomes of wheat. **Light green colour indicates low R**^**2**^ **values whilst dark blue indicates high values.** Data for observed genotypes are above the diagonal and genotypes simulated with gene dropping are below the diagonal. Markers are ordered according to position on the genetic map [20]. LD, linkage disequilibrium; SNP, single nucleotide polymorphism.

**Fig 7:**
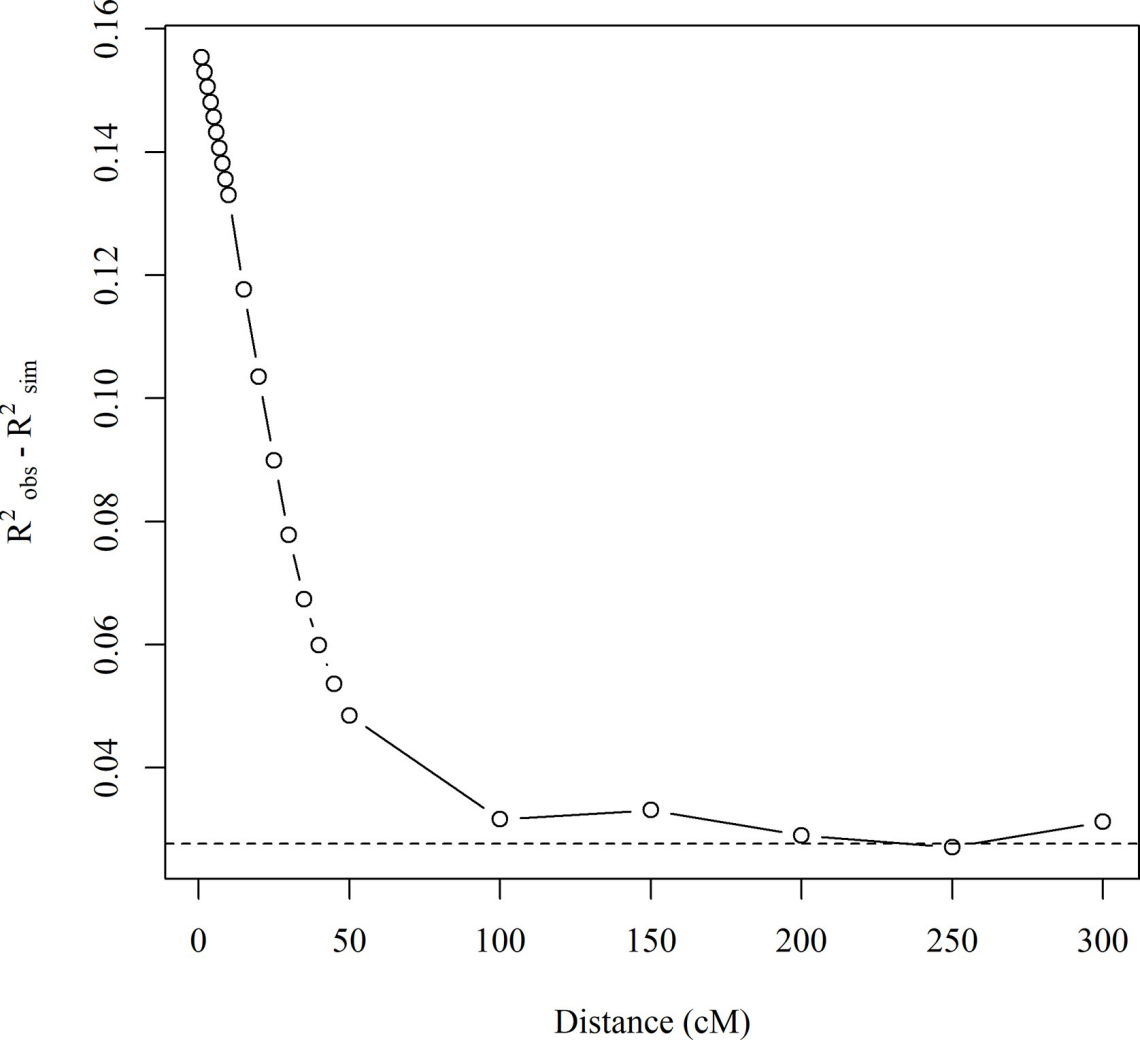
Relationship of the difference between observed and simulated LD against genetic distance of 790 SNP markers across all chromosomes. The horizontal dashed line indicates the average linkage between markers on different chromosomes. LD, linkage disequilibrium; SNP, single nucleotide polymorphism.

Selection can also be inferred from differences in LD decay between observed and simulated genotypes. Pairwise comparisons of LD were found to be much higher in observed than simulated data, across all chromosomes (Figs 6 and 7). For pairwise LD, the average genetic distance at which R^2^ fell to 0.15 was 25.9 cM in the observed data, compared to 11.2 cM for the simulated data (S2 Table). Furthermore, the magnitude of difference between observed and simulated data was highly dependent on the cM distance between markers. Average R^2^_obs_−R^2^_sim_ is constant at around 0.03 at genetic distances greater than 100 cM and between markers on different chromosomes but linearly increases to 0.16 as the distance between markers decreases from 50 cM to 1 cM (Fig 7). Collectively, this appears to demonstrate that directional selection by breeders has resulted in conservation of haplotype blocks containing beneficial gene combinations.

## Discussion

We developed a large pedigree database of wheat varieties, which spans over a century of breeding and variety development. The pedigree focuses on UK varieties but also includes accessions from 37 additional countries. To our knowledge, this is the largest integrated crop pedigree of its kind available as a complete pedigree for download and further inspection. The utility of the pedigree was demonstrated by comparing kinship coefficients calculated using SNPs as well as pedigree data and via gene dropping simulations to demonstrate significant selection by breeders over time.

The pedigree illustrates the significant landmarks in over 150 years of wheat breeding, from the simplest selections from regional heterogeneous landraces to the development of modern elite varieties. Recently developed software such as Helium [15] facilitates navigation, visualisation, and analysis of large-scale pedigrees such as that presented here. The degree of historical connections between varieties from different geographic backgrounds is notable and highlights the wide genetic background of UK wheat varieties. The ancestry of modern UK varieties typically includes varieties from France, Germany, the US, the Netherlands, Canada, and Sweden. When the ancestry is traced back as far as possible, UK varieties are shown to have commonly originated from a combination of landraces from various regions, including the UK, France, Germany, Scandinavia, Russia, the Mediterranean, Eastern Europe, India, and Mexico. However, the extent to which breeders are directly using parent varieties from different countries of origin has decreased in recent decades (S6 Fig). This practice was more common before the turn of the 20th century when intensive plant breeding programmes were initiated, as well as during the 1960s when many varieties were developed by state breeding programmes, such as the Plant Breeding Institute (PBI) in the UK, the Institut national de la recherche agronomique (INRA) in France, and CIMMYT in Mexico. As an example, in 1962, the French variety ‘Capelle Desprez’ was grown on 84% of the UK wheat growing area and was used extensively as a parent by the PBI for breeding in that decade [21]. Although the use of breeding material from different countries is less common now, this reflects a trend of increasing geographic adaptation of the UK wheat gene pool and a redistribution of the wide genetic basis within countries. However, recent developments in prebreeding approaches may have replaced geographic exchange of material as a source of breeding diversity, instead introducing genetic diversity that has never been present in the bread wheat gene pool. For example, the variety ‘Robigus’ includes putative novel *T*. *dicoccoides* introgressions [20], has been regularly used as a parent in UK elite varieties, and therefore makes a large contribution to modern UK genetic diversity. This also concurs with studies finding no reduction in genetic diversity over time due to breeding [22–23] and is contrary to commonly expressed concern over the effect of breeding activities on genetic diversity [24–25]. White and colleagues [26] also suggested that marker diversity in UK wheat varieties increased with the greater number of private breeding companies operating in the market since the PBI was privatised in 1987. Integrated information on pedigrees and kinship relationships, such as that presented here, will facilitate prioritisation of varieties as breeding material for management of these resources.

### Kinship comparisons

Using the entire pedigree, more than 121 million kinship coefficients were calculated. A subset of these were compared, with over 110,000 kinship coefficients also calculated using SNPs. The relatively high correlation between kinship calculations validates the pedigree and is much greater than coefficients previously found in smaller studies. Laidò and colleagues [27] found coefficients of 0.21 and 0.23 between pedigree kinships and Diversity Array Technology (DArT) or simple sequence repeat (SSR) markers, respectively, for a set of 116 durum wheat varieties; Soleimani and colleagues [12] found a coefficient of 0.46 for a set of 13 durum wheat varieties. In this study, the greater value of 0.71 could be due to the much larger size of the pedigree used, which spans a wide range of geographic origins and histories. This allows for a more complete comparison of pedigree kinships, as common ancestors between distantly related accessions are more likely to be identified. The weakly curvi-linear shape of the correlation found here (Fig 3) is caused by a wide range of marker kinship values at very low pedigree kinship values. This reflects low pedigree information in this region of the graph, i.e., missing pedigree data in parents, grandparents, and/or great grandparents for many relatively modern varieties in which unconnected founder individuals are assumed to be completely unrelated. An example of this is ‘Robigus’: although it has been commonly used in the pedigree of recent UK elite varieties, both of its parents are of unknown origin. Therefore, ‘Robigus’ and all of its descendants are subject to an underestimated pedigree-based kinship estimate. The same explanation may partly underlie the wider spread of marker kinship estimates above the diagonal line than below. However, on closer inspection, some of the more extreme upward outliers with marker kinship >0.9 appear to result from difficulty in correctly calculating pedigree kinship in complex older PBI and CIMMYT pedigrees that often included multiple backcross generations. Nevertheless, extreme deviations from the diagonal in either direction were highly informative in detecting errors, either in the published pedigree information or the material used for genotyping (S1 Text). The high proportion of comparisons with pedigree-based kinship close to 0.5 represent varieties with the same parentage.

Whilst the advantage of a pedigree-based approach to estimating kinship over a marker-based approach is that a much larger number comparisons can be made without the cost of genotyping, marker-based kinships provide a more informative estimate of genomic relationships for use in practices such as genomic selection [28–29] and QTL mapping studies [30]. In most species, marker relationship matrices are commonly used for trait prediction and association mapping in preference to using the pedigree. However, if marker densities are low or no markers are available, pedigree relationships continue to be used. A recent example in which both wheat marker- and pedigree-based estimates of kinship were fitted simultaneously found the inclusion of the pedigree improved the accuracy of trait prediction [31]. It is becoming more common to fit multiple estimates of relationship, e.g., derived from partitions of a marker set into separate classes [32], or for additive and dominance effects (e.g., [33]). It is pragmatic to include both matrices if available, and their relative merits will be decided within the analysis. An alternative option, not studied here, would be to combine the two so the markers could improve relationship estimates among founders, which are otherwise treated as unrelated, and the pedigree could help estimate relationships among individuals with missing marker data.

### Selection within crosses

One of the key assumptions of the pedigree-based approach to calculating kinship is that inheritance is random and in the absence of selection [34]. However, strong selection for traits, including improved yield, height, quality, and disease resistance, has undoubtedly taken place in wheat breeding programmes to achieve the genetic gains over the last century [35]. Here, we tested this assumption using a combination of the pedigree and genetic marker data to perform gene dropping simulations to compare observed variety genotypes against simulations in the absence of selection. By investigating the genetic relatedness of a set of varieties to each of their parents, we found that the majority of varieties demonstrated unequal parental contributions far outside the distribution predicted by simulations. This indicates that whilst the initial F_1_ from a breeder’s cross will carry exactly half of the alleles from each parent, the subsequent generations of inbreeding and segregation are opportunities for breeders to select segregants with a greater proportion of beneficial alleles that would have come from the superior parent. Our results support this and highlight the effectiveness and intensity of selection performed in wheat breeding programmes over and above simulated genetic drift. We also found that varieties that have been used extensively as parents, such as ‘Robigus’ and ‘Capelle Desprez’, are also favoured as the dominant parent in subsequent selections. This underlines the historic importance of these varieties and their contribution of beneficial genetic resources to advances in wheat breeding.

Our results also have implications for definition of essentially derived varieties (EDVs), defined by the International Union for the Protection of New Varieties of Plants (UPOV) as when ‘(i) it is predominantly derived from the initial variety, or from a variety that is itself predominantly derived from the initial variety, while retaining the expression of the essential characteristics that result from the genotype or combination of genotypes of the initial variety, (ii) it is clearly distinguishable from the initial variety and (iii) except for the differences which result from the act of derivation, it conforms to the initial variety in the expression of the essential characteristics that result from the genotype or combination of genotypes of the initial variety’ [36]. We found that wheat varieties derived from biparental crosses commonly share over 80% of their genetic material with one parent, which is greater than would be expected by backcrossing to a recurrent parent. This highlights the difficulties in defining a threshold of genetic similarity for EDVs in wheat and supports similar findings in other crops [37–38]. An alternative explanation for greatly differing parental relatedness would be that many registered varieties have been derived from incorrectly declared backcrosses. However, this is improbable given the median proportion of genome inherited from the maximally related parent was 0.57, which is substantially below the expected 0.75. Because it is uncommon for wheat varieties to be derived by backcrossing, especially with more modern varieties, we believe the observed excess distortion in favour of one parent is likely due to selection.

### Selection over the pedigree

We demonstrated selection over multiple generations by comparing observed and simulated genotypes in a subset of the pedigree in which 110 genotyped varieties released before the year 2000 were used to predict the distribution of marker alleles in the absence of selection of 45 varieties released after the year 2000. Amongst these 45 varieties, significant deviations from the expected simulated allelic diversity were used as an indication of breeders’ selection. When directional selection is in favour of one allelic variant, allelic diversity is ultimately reduced. Increased diversity could indicate selection for a more equal balance of alleles in the population, which may be the case if different alleles are favoured in contrasting wheat classes, such as for yield or quality. Alternatively, it could result from a transient polymorphism resulting from a rare allele, which has increased in frequency under selection but has yet to be fixed. Our results indicate that whilst average allelic diversity across all markers was similar to expected from simulations, a small number of lower than expected values were found for individual makers. This gives an indication of the location of genomic regions consistently under selection during the development of varieties released in the 21st century. The regions identified at the stringent significance thresholds used here are not localised to the known major flowering time loci *VRN-A1*, *VRN-B1*, *VRN-D1* (on the group 5 chromosomes; reviewed by [39]) or the dwarfing genes *RHT-B1* and *RHT-D1* (chromosome 4B and 4D, respectively). This is expected, because these loci of major phenotypic effect are already fixed in the materials and time spans used here to investigate selection. However, at the genetic resolution currently available, the major yield QTL on 7AL (*Qyld*.*csdh*.*7AL*) is thought to be principally due to increased grain number per ear [40–41] approximates to the 7A region of decreased diversity identified here, indicating selection for greater yield potential. This possible colocalisation is based on the IWGSC RefSeq version 1.0 wheat physical map positions of the peak 7A marker WMC273 identified by [40] and SNP IAAV5268 identified in this study (717.079 versus 679.839 Mbp, respectively). Given that this genetic locus (i) was identified after the year 2000 (here we investigated selection in materials pre- and post-2000) and (ii) has alleles of relatively large phenotypic effect, it is likely that beneficial alleles have been strongly selected at this locus in recent years. This finding highlights the possibility that the additional loci we detected are also under breeder phenotypic selection, as well as supporting the use of this approach for the identification of the genomic regions underlying breeding targets in other crops.

It has been suggested that progress in wheat breeding has in part been a result of assemblage of beneficial linked epistatic gene interactions [42]. This is supported by our comparison of observed LD with that expected from gene dropping simulations. It is evident that LD at short genetic distances is considerably higher than expected from simulations. This suggests that breeder selection is favouring conservation of favourable haplotypes and linkage blocks. Rhoné and colleagues [43] found effects in an experimental wheat population grown under natural selection in which selection favoured an important yellow rust resistance gene, increasing LD and reducing diversity around the gene region. However, some of these effects may also be explained by, or be in addition to, (i) the strong segregation distortion around common putative introgression fragments identified in the genetic map used here (constructed using a multiparent mapping population in the absence of intentional selection) [20] or (ii) by inflated map distances. The implications of these findings go some way to explaining the relatively high levels of LD found in association mapping panels of highly selected varieties [26,44–45]. Methods are being developed to increase recombination in domesticated crops [46] because there is concern that there is insufficient recombination within the pericentromeric regions of domesticated crop genomes. The results here suggest that care is required: increased recombination in breeders’ germplasm will be beneficial if the extensive LD we have found is predominantly a result of linkage drag or hitchhiking but will be disadvantageous if it breaks up favourable linkages that may have been built up over many generations of selection. The difference between observed and simulated local LD estimates is much higher than that for long-distance LD. Nevertheless, long-distance LD is also weakly elevated in the observed versus simulated data (0.042 versus 0.0141 for off-chromosome averages; see Fig 6 for visual comparison). This may be partly generated by directional selection causing segregation distortion (Fig 4). However, scattered regions within which much higher than expected LD between markers on different chromosomes could be identified (e.g., between blocks on chromosomes 1D and 2B, Fig 6) likely indicate selection for more distant epistatic genetic relationships or directional selection on polygenic traits [47].

### Detecting selection: Comparison to previous approaches

Pedigree-based tests for selection are more common in animals and humans than in plants. Our test for selection within families is essentially the same as the transmission disequilibrium test [48–49], which can be regarded as a test for the efficiency of selection on progeny in distorting segregation patterns from the parents. In the plant equivalent, no distortion is possible at the F_1_, provided the parents are homozygous, but we may detect distortion after several subsequent generations of selfing (or doubled haploid production) with accompanying selection. Because we do not have large numbers of simplex families, tests for distortion at individual loci would have low power. However, we have found extensive distortion in favour of one of the parents in many crosses.

Larkin and colleagues [50] detected selection in dairy cattle by using approximately 1 million SNPs to reconstruct haplotypes of two elite bulls and comparing observed and expected frequencies in 1,149 descendants. They found 49 chromosome segments with strong evidence of selection. Due to the current limit in available wheat genotyping array SNP densities, our data are less extensive, but our approach of using gene-dropping simulations in the pedigree is similar in exploiting the pedigree. Recent work in cattle also suggests that for highly polygenic traits in which selection intensity on any individual locus may be weak, detecting selection in pedigrees may be more appropriate than alternative population based methods, which can have many confounding effects [51]. Here, we have used the available data to illustrate the utility of reconstructing the whole wheat pedigree.

### Further development

We present a pedigree resource for wheat varieties released in the UK up to 2017. The resource is available at https://www.niab.com/pages/id/501/UK_Wheat_varieties_Pedigree, where it will be periodically updated to incorporate newly released varieties. It is anticipated that further engagement with the wheat breeding community will enable correction of errors in historic pedigrees, as well as provision of pedigree data that is not yet publicly available, thus augmenting the utility of the resource. Whilst the pedigree focuses on UK wheat varieties, significant historic crossover with varieties originating from other countries will facilitate interlinking of fragmented wheat pedigrees maintained in other countries or breeding companies and extend the utility of the resource to the wheat breeding community across the world. Future analysis of the current pedigree will focus on identifying selection on haplotypes.

### Conclusions

We present a comprehensive wheat pedigree as a resource for the wheat research and breeding community. Recently developed software enables visualisation and navigation of the pedigree, as well as highlights historically important varieties and the diverse origins of elite UK wheat varieties. Comparison of kinship coefficients calculated using the pedigree, as well as genetic markers, validated the pedigree and allowed identification and correction of pedigree and genotyping errors. In conjunction with pedigree and genotypic data, gene dropping simulations demonstrated significant effects of selection within crosses as well as over multiple generations of the pedigree, modulating allelic diversity and conserving LD. These analyses identify the genomic regions controlling putative wheat breeding targets and serve as a model for the identification of genomic regions controlling breeding targets in other crops. The resource developed here will serve as an evolving platform to inform and manage wheat genetic diversity in breeding programmes in the UK and around the world and highlights the need to develop and maintain similar resources in other crop species.

## Materials and methods

### Data collection and visualisation

Pedigree information was sourced from publicly available breeders’ records, genebank passport information (http://genbank.vurv.cz/ewdb/), associated information with commercial varieties released in the UK (https://cereals.ahdb.org.uk/varieties/ahdb-recommended-lists.aspx), textbooks [21,52], wheatpedigree.net (and references therein), and with permission, from breeder’s private records. Genotypic data, available for 454 accessions within the pedigree, were previously generated within the Biotechnology and Biological Sciences Research Council (BSBRC) project Wheat Association Genetics for Trait Advancement and Improvement in Lineages (grant reference BB/J002542/1), using the wheat 90k Illumina iSelect SNP array [53], following previously described methods [54]. The raw genotype data were sourced from http://www.niab.com/pages/id/326/Resources/. Pedigrees were visualised using Helium version 1.18.03.15 [15].

### Calculation and comparison of kinship coefficients

Pairwise comparisons of kinship based on the pedigree were calculated using the ‘kinship2’ package [55] in R [56]. The pedigree was augmented to include seven intermediate generations of selfing from parents to progeny for each accession to account for multiple generations of inbreeding used in wheat variety development. This is, of necessity, an approximation and cannot account for lines created from doubled haploids (information for which is frequently not available). The kinship coefficient between two individuals is the probability that a randomly selected allele at a locus in one individual is identical by descent with a randomly selected allele in the other. Individuals with no known common ancestors in the pedigree have a kinship of 0. Fully inbred sibling lines of inbred parents have a kinship of 0.5, and kinship of an inbred line with itself is 1.

Kinship coefficients based on genetic markers were calculated based on identity by state (IBS) allele-sharing implemented in R/EMMA (http://mouse.cs.ucla.edu/emma/) for the 454 accessions that were in common with the pedigree. An IBS approach was chosen for simplicity and ease of comparison with the pedigree estimates, because the assumption that alleles are drawn from a random global population—and the subsequent estimation of allele frequencies—is problematic for calculating genomic relationship matrices in the complexly structured population of inbred lines found in the pedigree. The 26,018 available polymorphic Illumina iSelect SNPs were thinned to remove closely linked markers by removal of one of each pair of markers with an absolute correlation >0.75 to minimise the effect of the very high levels of marker clustering observed in cereal crops [57], resulting, e.g., from commonplace interspecific introgressions [20] and large nonrecombining tracts spanning the centromeres [58]. This resulted in 4,009 SNPs for downstream analyses.

A comparison of pedigree-based and marker-based kinship coefficients was made for the 102,831 pairwise comparisons between accessions with both pedigree and marker data, by calculating the Pearson correlation coefficient between elements of the two kinship matrices. To confirm whether an appropriate number of generations of inbreeding was used to estimate the pedigree kinships, the comparison between pedigree and marker based kinships was also made with pedigree kinships based on 5, 7, and 10 generations of inbreeding. Correlation coefficients with marker based kinships differed on average by only 0.02%.

### Selection within families

Of the 454 genotyped accessions, 109 also had genotype data for both parents. For each of these ‘simplex families’ (i.e., two parents and their one progeny), the proportion of alleles inherited from each parent was calculated. To estimate the variation in this measurement in the absence of selection, Genedrop (NIAB, Cambridge, UK) simulation software (as described in [19]) was used. Briefly, gene dropping is a permutation analysis, in which multiple simulations are run assuming mendelian inheritance with a 50:50 transmission probability of alleles from parent to offspring of genetic loci defined in the founder generation. The frequency of alleles in the multiple repeated simulations enables definition of probabilities of the occurrence of observed genotypes. Following common practice, we use the term ‘gene dropping’ here to describe the simulation of the descent of multiple loci, with recombination (based on a provided genetic map), through a pedigree, and not just the inheritance of a single locus. One thousand simulations were run using two parents that were polymorphic at all 4,009 loci so that the F_1_ between the parents was completely heterozygous. SNP genetic map positions were sourced from the eight-parent ‘NIAB Elite MAGIC’ genetic map [20]. Seven generations of selfing were included to account for inbreeding in the variety development process. The proportion of alleles inherited from each simulated parent was calculated for each simulation and *P* = 0.01 and *P* = 0.001 significance thresholds determined from this empirical distribution.

### Selection across the pedigree

To investigate selection effects over multiple generations, a subset pedigree was made that included 110 founding accessions with genotype data that were released before the year 2000. This date was chosen as the approximate median value of the date of release of the varieties with available genotype data. From these genotyped founders released prior to 2000, 207 descendants could be identified, which do not include ancestry outside of the founder gene pool. Forty-five of the descendants were released after the year 2000 and had genotypic data. One hundred simulations were carried out for this pedigree subset using Genedrop software with the 1,821 polymorphic SNPs available with genetic map positions sourced from [20]. Markers were selected to remove one of each pair of markers with an absolute correlation >0.75 or that mapped to the same position on the ‘NIAB Elite MAGIC’ genetic map [20]. As with other simulations, seven assumed generations of selfing were included to account for inbreeding.

From the 45 descendants, estimates of allelic diversity were calculated for each biallelic marker locus for both observed and simulated genotype data. Diversity [59] was calculated as

$$1-\sum \left(p_{i}^{2}\right),$$ (1)

where *p*_i_ is the frequency of the *i*th allele.

Squared correlation coefficients (R^2^) were calculated for all 311,655 pairwise comparisons among 790 markers across the genome for observed and simulated data to estimate LD. These markers were a subset of the 1,821 outlined above with a minor allele frequency >0.2. For each chromosome, the simulated and observed LD decay curve was modelled using a loess curve fit, with span smoothing parameter set to 0.75. The fitted curve was used to estimate the genetic distance at which LD fell to 0.15 for each chromosome in the observed and simulated data sets. The fitted curve was additionally used to estimate the average LD at different genetic distances along the chromosomes.

### Anchoring genetic markers to the wheat genetic map

To anchor genetic markers to the physical map, DNA sequences associated with selected genetic markers were used as queries for BLASTn (University of Washington, WA) [60] searches of the wheat cv ‘Chinese Spring 42’ genome assembly (IWGSC RefSeq version 1.0 [58]), and the hits were ranked by expectation value (*e*-value).

## Supporting information

S1 Fig High resolution image of Fig 1. (PNG)

S2 Fig The forward pedigree of the wheat landrace, ‘Red Fife’, illustrating the extent a single landrace selection (i.e., a very old variety) has contributed to the geneology of modern wheat varieties. (PNG)

S3 Fig High resolution image of Fig 2. (PNG)

S4 Fig Relationship between genetic and pedigree based kinships coefficients amongst 454 wheat varieties using 4,009 SNP markers. Points in red indicate kinship comparisons involving the varieties ‘Cyber’ and ‘Maris-Ensign’, for which the kinship and pedigree relationship estimates place the varieties in different major wheat populations (‘winter’ versus ‘spring’).
(TIF)

S5 Fig A subset pedigree consisting of genotyped founders released before the year 2000 (red), and derived varieties with genotype data released after the year 2000 (yellow). Node size is proportional to number of direct offspring in the subset.
(TIF)

S6 Fig Histogram illustrating how the percentage of variety parents with a different country of origin to the derived variety varies over decade. (TIF)

S1 Table The wheat pedigree (v11.10.18), consisting of 2,657 entries, formatted for use in the software, Helium [15]. Additional information (year of release and country of origin) is also presented in Helium format.
(XLSX)

S2 Table Differences in observed versus expected LD for each of the 21 wheat chromosomes. Expressed as distance (cM) when linkage R^2^ equals 0.15 from fitted loess curves for observed and simulated genotypes. Summary statistics shown are per chromosome, per subgenome, and across all chromosomes. LD, linkage disequilibrium.
(DOCX)

S1 Text Detecting errors in the published pedigree. (DOCX)

## Abbreviations

BSBRC: Biotechnology and Biological Sciences Research Council
CIMMYT: International Maize & Wheat Improvement Center
DArT: Diversity Array Technology
EDV: essentially derived variety
IBS: identity by state
INRA: Institut national de la recherche agronomique
LD: linkage disequilibrium
PBI: Plant Breeding Institute
QTL: quantitative trait locus
SNP: single nucleotide polymorphism
SSR: simple sequence repeat
UK: United Kingdom
UPOV: International Union for the Protection of New Varieties of Plants
