# Supplementary figures and images for: A large-scale pedigree resource of wheat reveals evidence for adaptation and selection by breeders
Source: PLoS Biol. 2019 Feb 28;17(2):e3000071. doi: 10.1371/journal.pbio.3000071 (PMC6413959; doi:10.1371/journal.pbio.3000071)

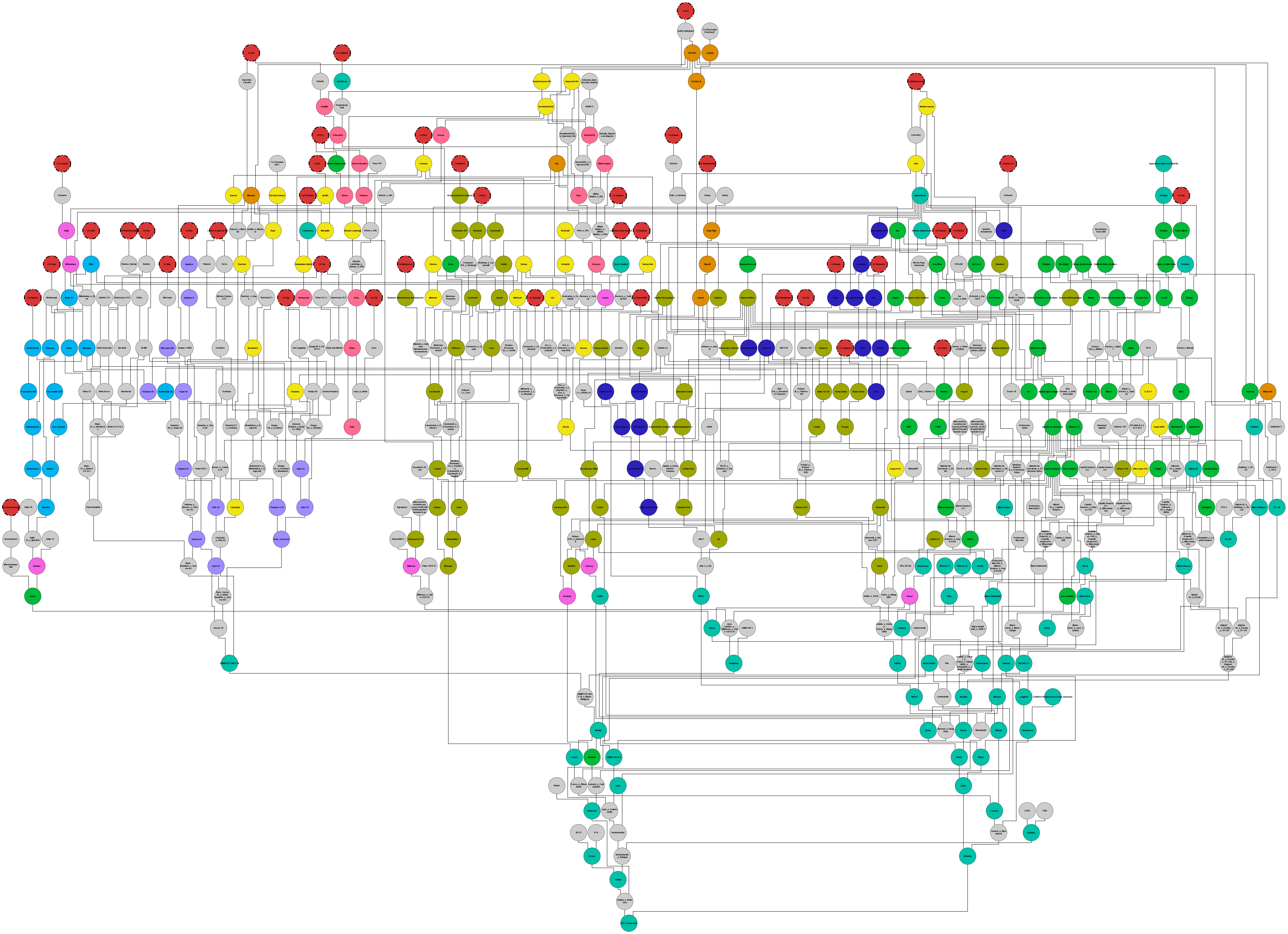

Supplement: S3 Fig — (PNG) [file pbio.3000071.s003.png]

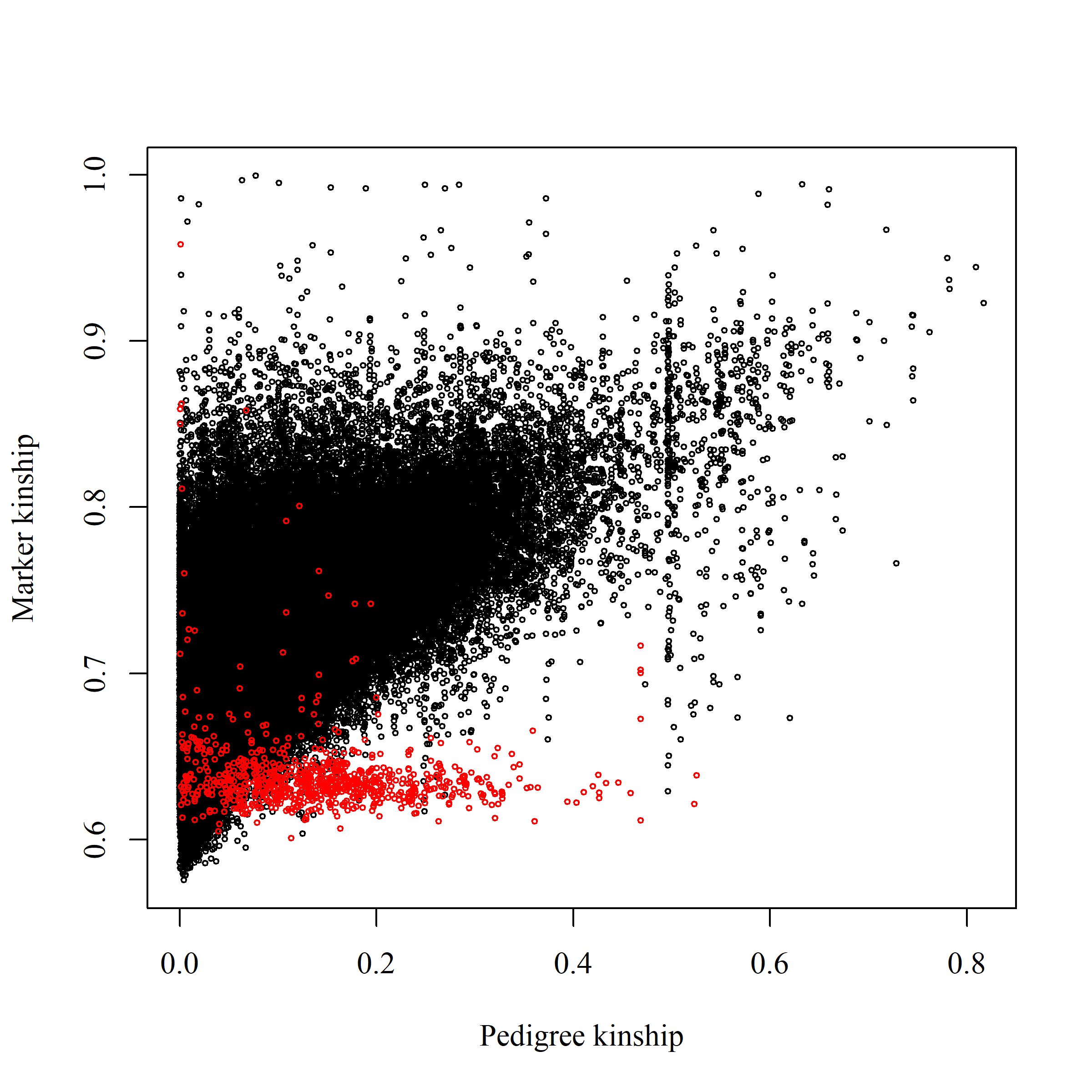

Supplement: S4 Fig — Points in red indicate kinship comparisons involving the varieties ‘Cyber’ and ‘Maris-Ensign’, for which the kinship and pedigree relationship estimates place the varieties in different major wheat populations (‘winter’ versus ‘spring’). (TIF) [file pbio.3000071.s004.tif]

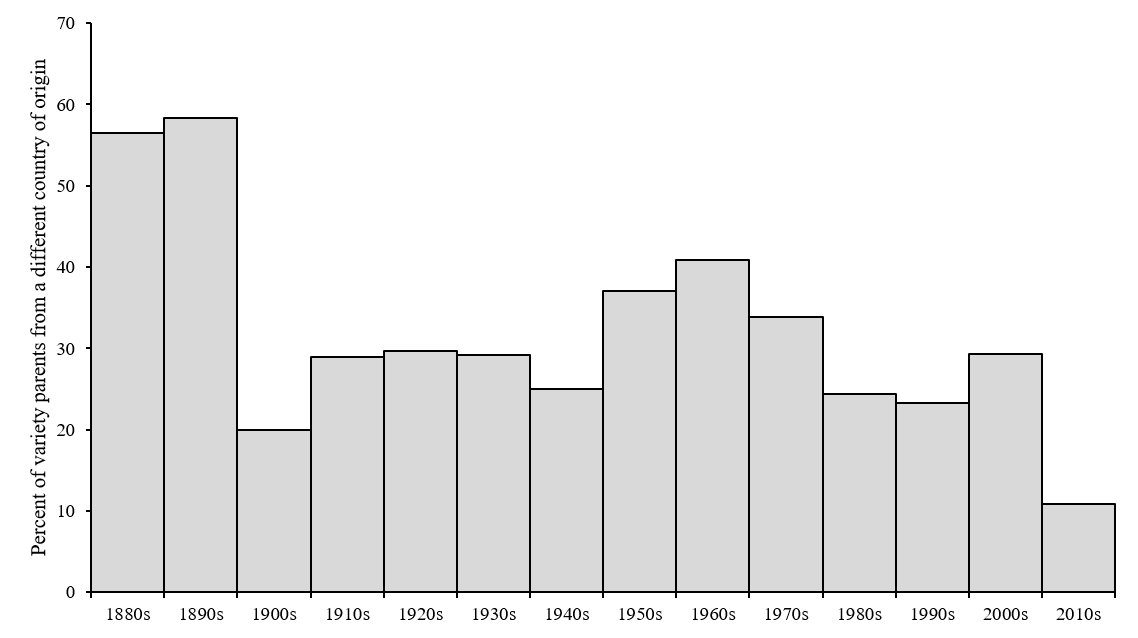

Supplement: S6 Fig — (TIF) [file pbio.3000071.s006.tif]
