# Supplementary material for: A large-scale pedigree resource of wheat reveals evidence for adaptation and selection by breeders
Source: PLoS Biol. 2019 Feb 28;17(2):e3000071. doi: 10.1371/journal.pbio.3000071 (PMC6413959; doi:10.1371/journal.pbio.3000071)
Supplement: S2 Table — Expressed as distance (cM) when linkage R2 equals 0.15 from fitted loess curves for observed and simulated genotypes. Summary statistics shown are per chromosome, per subgenome, and across all chromosomes. LD, linkage disequilibrium. (DOCX) [file pbio.3000071.s008.docx]

|  | **Distance (cM) when R^2^ = 0.15** | |
| --- | --- | --- |
| **Chromosome** | **Observed** | **Simulated** |
| 1A | 26.3 | 10.9 |
| 1B | 24.2 | 2.1 |
| 1D | 13.4 | 7.6 |
| 2A | 19.3 | 4.9 |
| 2B | 47.0 | 12.2 |
| 2D | 20.0 | 10.5 |
| 3A | 29.1 | 11.7 |
| 3B | 23.7 | 3.4 |
| 3D | 20.1 | 18.1 |
| 4A | 15.8 | 8.1 |
| 4B | 38.3 | 12.3 |
| 4D | 16.9 | 12.0 |
| 5A | 26.2 | 8.7 |
| 5B | 37.5 | 19.5 |
| 5D | 18.7 | 15.7 |
| 6A | 27.0 | 14.8 |
| 6B | 28.1 | 11.2 |
| 6D | 20.0 | 14.0 |
| 7A | 38.0 | 9.4 |
| 7B | 28.6 | 10.7 |
| 7D | 25.7 | 18.4 |
| All | 28.1 | 10.0 |
| Average | 25.9 | 11.2 |
| All A genome | 25.9 | 9.8 |
| All B genome | 32.5 | 10.2 |
| All D genome | 19.3 | 13.8 |

**S2 Table. Differences in observed versus expected linkage disequilibrium (LD) for each of the 21 wheat chromosomes.** Expressed as distance (cM) when linkage R^2^ equals 0.15 from fitted loess curves for observed and simulated genotypes. Summary statistics shown are per chromosome, per subgenome, and across all chromosomes.
