# Supplementary material for: A large-scale pedigree resource of wheat reveals evidence for adaptation and selection by breeders
Source: PLoS Biol. 2019 Feb 28;17(2):e3000071. doi: 10.1371/journal.pbio.3000071 (PMC6413959; doi:10.1371/journal.pbio.3000071)
Supplement: S1 Text — (DOCX) [file pbio.3000071.s009.docx]

**S1 Text.** Detecting errors in the published pedigree.

For example, the variety ‘Aardvark’ was found in many outlier comparisons because it seemed unrelated by markers kinship to its reported ancestors or descendants, yet its ancestors and descendants were related to each other as would be expected if they shared ‘Aardvark’ as a relative. This strongly suggested that the seed source used to genotype Aardvark was wrong. More visibly, the disproportionate number of comparisons with marker based kinships of approximately 0.63 that had a higher than expected pedigree based kinship (highlighted in S2 Fig) were all due to two varieties, ‘Cyber’ and ‘Maris Ensign’, which were both misclassified as spring or winter wheats, respectively.
